# Marketing technology in macroeconomics

**DOI:** 10.1186/2193-1801-1-28

**Published:** 2012-10-04

**Authors:** Kenichi Tamegawa

**Affiliations:** School of Commerce, Meiji University, 1-1 Kanda-Surugadai, Chiyoda-ku, Tokyo, 101-8301 Japan

**Keywords:** DSGE modeling, Marketing, Matching friction

## Abstract

In this paper, we incorporate a marketing technology into a dynamic stochastic general equilibrium model by assuming a matching friction for consumption. An improvement in matching can be interpreted as an increase in matching technology, which we call marketing technology because of similar properties. Using a simulation analysis, we confirm that a positive matching technology shock can increase output and consumption.

## Introduction

The considerable progress in information technology (IT) since the late 1990s increased the productivity of goods and contributed to the IT boom in the economies of many countries in the 2000s. In economics, IT development is typically expressed as an increase in total factor productivity (TFP). This is a point of view from supply side of the economy. [Jorgenson (2001]) and Jorgenson et al. ([2008]) pointed out that nonfarm business productivity growth surged from 1997 to 2001.

**Figure 1:**
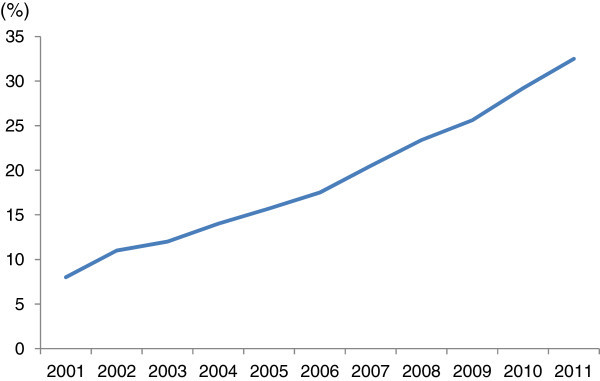
**Internet users across the globe.** Note: The line depicts percentage per 100 inhabitants. Source: ITU World Telecommunication/ICT Indicators database.

In addition to the supply side effects of IT, we can consider that IT also affects the demand side. Through web sites such as Amazon.com, for example, the Internet enables us to buy numerous goods instantaneously. A recent development in IT, the so-called Web 2.0, which includes social networking services such as Facebook, has enabled firms to contact individual consumers and promote their products. Recent developments in mobile phone technology, for example the iPhone, provide opportunities for matching consumers and products. This is reflected in the worldwide increase in Internet users (see Figure 1), which in turn increases opportunities for matching. In convenience, we call the technology, which enhances matching opportunity, “marketing technology,” because it can easily match consumer needs with a firm’s products and therefore resembles the concept of marketing. Broadly speaking, IT may enhance productivity as stated above. In this paper, however, we limit the scope of marketing technology to that which provides greater opportunities for sales, since studies for supply side technology like TFP are plentiful.

The way that production technology affects business cycles is well known^1^, but research on the effects of technology such as marketing technology on the macroeconomy has not yet been undertaken at least within the framework of macroeconomics. It is therefore quite interesting to investigate the effects of marketing technology. Our goals are as follows: first, to incorporate the marketing sector into an economic model; second, to assess the effects of the positive shock of marketing technology on the macroeconomy. First of all, we express marketing technology in an economic model by employing a matching or search friction in the goods market. Researchers have frequently employed this assumption in the labor market on the basis of [Mortensen and Pissarides (1994]). Adopting the matching friction suits our purpose because progress in marketing technology can be modeled as a reduction of matching friction between consumers and firms.

To accomplish the second goal, we use a dynamic stochastic general equilibrium (DSGE) model, which is a useful tool in analyzing the macro economy. The model consists of identity equations and behavioral equations that are derived from agents’ optimization problems^2^. Our model is constructed on the basis of a standard real business cycle (RBC) model as described in King et al. ([1988])^3^. Of course, it can easily be extended to a New Keynesian model by adding a sticky price assumption, as used in Christiano et al. ([2005]).

In this paper, we show the effects of marketing technology by performing a numerical simulation. The main result is a positive response of output, which occurs because progress in marketing technology can increase matched consumption. In our settings, the sudden increase in households’ consumption provides an incentive to work more to smooth out the consumption path. Similar to increases in TFP, developments in IT technology that affect the demand side can also increase output and therefore income.

The remainder of our paper is organized as follows. Section 2 explains the key equation, which plays an important role in this paper. Section 3 constructs our model. Section 4 presents a simulation analysis of marketing technology. Section 5 discusses how incorporating the marketing sector into a DSGE model alters the model’s responses to shocks from other marketing technologies. Section 5 concludes the paper.

## Matching friction for consumption

This section explains the key equation of our model: matching friction. Suppose that a consumer has a consumption plan, denoted by C_t_, and that firms use some amount of resources denoted by *a*_*t*_ to advertise their goods. We then assume that consumer needs are met through the following Cobb-Douglas type matching function:1

where *C*_*t*_^*m*^ represents matched consumption. In the above equation, an increase in *Z*_*t*_^*C*^ implies that the matching opportunity becomes bigger. We therefore call it marketing technology. High planned consumption and advertisement also facilitate the matching. The motivation of assuming Eq (1) stems from the study of matching friction for the labor market introduced by [Mortensen and Pissarides (1994])^4^. In their study, labor matching results from a combination of vacancies offered by firms and the labor force provided by households. This assumption is also useful in a consumption matching framework.

For the following simulation, we assume that log *Z*_*t*_^*C*^ follows an AR(1) process. Note that under this setting,  can be interpreted as a matching probability. Moreover, in Eq (1), If *γ =* 1 and *Z*_*t*_^*C*^ ≡ 0, the model constructed below is reduced to a standard RBC model.

## Model

In our model, firms have a marketing sector and a production sector, households live infinitely, and there exists a the government. The population is normalized to 1. We begin by explaining the matching friction.

### Firms: Production sector

Firms in the production sector have the following Cobb-Douglas production function:2

where *Y*_*t*_ represents output; *K*_*t*_, capital stock; *h*_*t*_, hours worked; And log *Z*_*t*_^*Y*^, a productivity shock with mean 0. With this technology of production, firms’ gross profits are as follows:3

where *a*_*t*_ is the goods used in advertising. The net output for firms is therefore *Y*_*t*_ − *a*_*t*_. The first-order condition for profits maximization yields4

The gross rental rate is as follows:5

### Firms: Marketing sector

The marketing sector receives *a*_*t*_ from the production sector and conducts marketing activities. Consequently, their goods meet consumer needs through the consumption matching function. As stated above, to conduct this activity, we assume that the marketing sector needs *a*_*t*_. The marketing sector demands *a*_*t*_ to maximize *C*_*t*_^*m*^ − *a*_*t*_. The first-order condition is6

Note that *C*_*t*_^*m*^ − *a*_*t*_ is not profit but merely a hypothetical objective function.

### Households

This subsection explains the aggregated behavior of households. First note that households are subject to the following inter-temporal budget constraint^5^:7

where *D*_*t*_ represents financial assets and *T*_*t*_ denotes lump-sum tax. Assuming that temporal utility is log *θ*_*t*_*C*_*t*_, households decide their planned consumption and labor supply by maximizing the following utility function, given {θ_t_}:

where *β* represents a discount rate. The first-order conditions are89

Note that the consumption path is independent {θ_t_} as shown in Eq (8).

### Equilibrium condition

Assuming that capital stock is accumulated As *K*_*t* + 1_ = (1 − *δ*)*K*_*t*_ + *I*_*t*_ with a depreciation rate of *δ* and an investment of *I*_*t*_ and that the primary balance for the government is always zero, an equilibrium condition *K*_*t+1*_ = *D*_*t+1*_ yields10

where *G*_*t*_ represents government expenditure (which is equal to *T*_*t*_).

**Figure 2:**
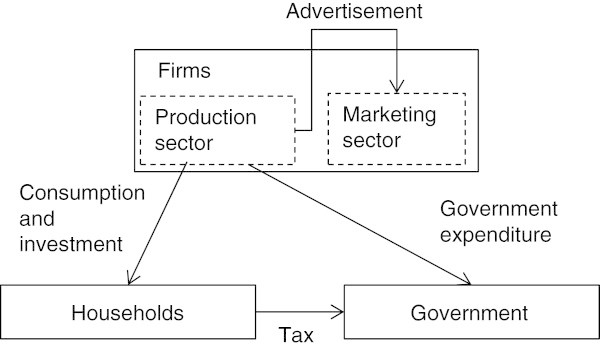
**Flow of Goods.**

For convenience of understanding the flow of goods, we provide Figure 2. First, firms produce goods using labor and capital goods that are provided from households. Households (consumers) consume the goods and pay tax to government. Advertisement is implemented through the goods that firms produce; in other words, advertisements are own consumption for firms.

## Simulation analysis

How does the model behave against a positive marketing shock? First, since this shock provides a matching opportunity, matched consumption increases; consequently, saving decreases. This decrease in turn raises the rental rate and provides an incentive to work more. Therefore, output also increases. Planned consumption nevertheless decreases because rental rate increases. Although a matching improvement increases output over several periods, consumption later decreases because of a consumption-smoothing motive. On the other hand, an increase in saving reduces the rental rate and causes a decrease in the labor supply also decreases. Intuitively speaking, an increase in matching technology raises consumption; this forces households to work more to compensate for the increased consumption. As a result, output increases.

**Figure 3:**
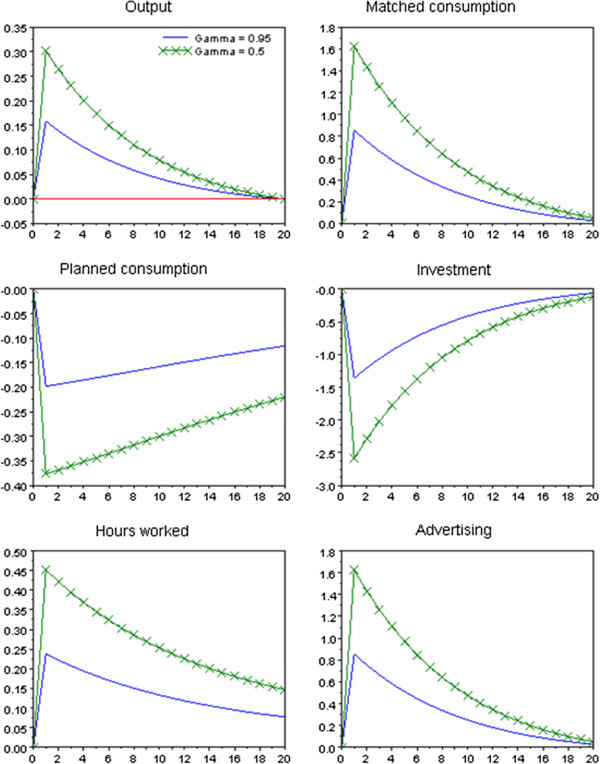
**Impulse responses to the marketing technology shock.** Note: The above lines are shown as the percentage deviations from the steady state.

To confirm the above theoretical conjecture, we linearize and simulate the model. The parameter settings are [*α β δ C*^*m*^*h*] = [1/3 0.99 0.02 0.6 1/3], where *C*^*m*^ and *h* denote the steady-state values. For γ, we consider γ = 0.95 and γ = 0.5. A persistency parameter for log *Z*_*t*_^*C*^ is 0.9. The output share of advertising is 0.01 in the steady state. In Figure 3, we show impulse responses to the one percent shock for log *Z*_*t*_^*C*^. In the case of γ = 0.5, since consumption matching is strongly affected by advertising, responses to the marketing shock are volatile.

As shown above, while a positive marketing shock can raise output, it decreases investment. This phenomenon seems to contrast with the experience of the late1990s. In the actual economy, however, IT can increase TFP. We can therefore consider that for this period, investment increases through a positive TFP shock. Of course, since there is a possibility that matching technology increase investment in the actual economy, careful empirical research is needed.

## Discussion: Consumption matching friction neutrality

How does the consumption matching friction alter responses to a supply or demand shock other than by matching technology relative to a standard RBC model? In a linearized model, the answer is that the friction does not alter the other shock responses. This is because households know how much their needs are matched by goods produced by firms; in other words, they know the matching probability *θ*_*t*_. Households then know the amount of goods to consume under a given shock even though matching friction is assumed. This implies that *C*_*t*_^*m*^ does not depend on the value of γ. Regardless of the value of γ, the responses to shocks other than the marketing technology shock are not altered.

This neutrality is not a drawback but an attraction from the empirical view point. Incorporating a consumption matching friction into a DSGE model may improve the results of empirical analyses such as that of [Smets and Wouters (2003]), since adding this assumption does not harm the model properties. Further, marketing technology is considered to be a new structural shock. With this new shock, the model can allow for richer dynamics, which helps reduce the problem of the degree of stochastic singularity (see Ruge-Murcia, [2007] and Tovar, [2009]).

## Concluding remarks

In this paper, we incorporated a marketing technology into a DSGE model by assuming a matching friction for consumption. The improvement in matching could be interpreted as an increase in matching technology. Using a simulation analysis, we confirmed that positive matching technology shock can raise output and consumption.

Further implications of what this paper has demonstrated in theoretical results need to be assessed through empirical studies. Fortunately, methods of empirical research on the basis of a DSGE model, for example, the method that [Smets and Wouters (2003]) used, are now becoming more familiar to economists. To investigate the effects of marketing technology on the economy of the late 1990s is quite interesting, but this is left for the future.

## Endnotes

^1^For example, see [Romer (2011]).

^2^The motivation for using the DSGE models in analyzing the macroeconomy is to avoid the famous critique by [Lucas (1976]): a model has to be described such that it is invariant to exogenous shock.

^3^Famous DSGE models are surveyed in [Tovar (2009]) and Mc[Candless (2008]).

^4^There are many studies that investigate the effects of labor market friction on business cycles. For example, see [Shimer (2010]).

^5^This expression of budget constraint can be archived from the law of large numbers for *θ*_*t*_.

## Abbreviations

DSGE: Dynamic stochastic general equilibrium
IT: Information technology
TFP: Total factor productivity
RBC: Real business cycle
AR: Auto regression.
